# Microwave Absorption and Magnetic Properties of M-Type Hexagonal Ferrite Ba_0.95_Ca_0.05_Fe_12−x_Co_x_O_19_ (0 ≤ X ≤ 0.4) at 1–18 GHz

**DOI:** 10.3390/ma17215327

**Published:** 2024-10-31

**Authors:** Juan Li, Hao Yao, Yuting Huang, Hongxia Wang

**Affiliations:** 1 College of Materials Science and Engineering, Zhejiang University of Technology, Hangzhou 310014, China; 2 Research Center of Magnetic and Electronic Materials, Zhejiang University of Technology, Hangzhou 310014, China

**Keywords:** hexagonal ferrite, M-type barium ferrite, Co_2_X phase, microwave absorption, magnetic property

## Abstract

In order to improve the microwave-absorption performance of barium ferrite and broaden its microwave-absorption band, BaFe_12_O_19_, Ba_0.95_Ca_0.05_Fe_12_O_19_, and Ba_0.95_Ca_0.05_Fe_12−x_Co_x_O_19_ (x = 0.1, 0.2, 0.3 and 0.4, respectively) hexaferrites were synthesized by the solid-state reaction method, and the influence of Co ion substitution on the phase composition, microstructure, magnetic properties, and microwave-absorption ability of the ferrites in this system was studied. Introducing minor Co ions (x < 0.2) facilitated sintering and grain growth. At x ≥ 0.2, XRD revealed the emergence of the Co_2_X phase alongside the BaM phase. Increasing Co ion concentration and the secondary X-phase led to slight reductions in saturation magnetization (69 to 63.5 emu/g) and substantial decline in coercivity (2107.02 to 111.21 Oe), attributed to grain size growth and Co_2_X’s soft magnetic nature. Notably, Co_2_X incorporation significantly enhanced the microwave absorption and provided a tunable absorption band from the Ku to the C band. For a sample with a thickness of 2.0 mm and a doping level of x = 0.2, a minimum reflection loss of −59.5 dB was achieved at 8.92 GHz, with an effective absorption bandwidth of 3.31 GHz (7.07–10.38 GHz). The simple preparation method and good performance make Ba_0.95_Ca_0.05_Fe_12−x_Co_x_O_19_ (x = 0.1, 0.2, 0.3 and 0.4, respectively) hexaferrites promising microwave-absorbing materials.

## 1. Introduction

As information technology advances, electronic devices functioning within the microwave and millimeter-wave frequencies are increasingly susceptible to electromagnetic pollution, a condition that can potentially result in electromagnetic interference, thereby causing malfunctions among interconnected electronic systems. Consequently, the development of microwave-absorbing materials, featuring substantial reflection loss and a broad bandwidth, has become a pressing concern in the field [1,2,3,4]. Among the myriad of materials explored, ferrites have garnered significant attention due to their low cost, high stability, and exceptional microwave magnetic-loss capabilities [5,6,7]. Since their inception, various crystal types of ferrites, including magnetite, spinel, and garnet, have been extensively studied for their exceptional chemical stability, substantial magnetic losses, and high resistivity. These attributes render them promising candidates as electromagnetic-wave absorbers and interference shielding materials for both commercial and military applications [8,9,10,11,12].

Within the family of ferrites, M-type barium ferrite (BaFe_12_O_19_), featuring a magnetite-like microstructure, exhibits noteworthy microwave-absorption capabilities attributed to its distinctive hexagonal plate morphology and substantial magnetic losses at gigahertz frequencies [13,14]. However, despite their proficiency in achieving significant losses at these frequencies, M-type barium ferrites may encounter challenges in achieving efficient absorption within specific frequency bands. This necessitates precise tuning of the material within these target bands, to optimize its absorption performance. This frequency-dependent behavior could potentially restrict their practical applicability across a broader frequency spectrum, particularly in scenarios demanding multiband wave-absorption capabilities. Consequently, enhancing the wave-absorbing properties of M-type ferrites across diverse frequency ranges to broaden their versatility constitutes a pivotal research objective.

To enhance the microwave-absorption capabilities of M-type barium ferrite (BaFe_12_O_19_) and align with the requirements of “thin, light, broad, and strong” wave-absorbing materials, various research endeavors have explored the impact of ionic substitutions at barium or iron sites on its microwave-absorption properties. For instance, Goel et al. employed the sol–gel auto-combustion method to synthesize lanthanum-doped hexagonal barium ferrite nano-particles (BaLa_x_Fe_12−x_O_19_) specifically tailored for X-band (8–12 GHz) absorption. Notably, BaLa_0.06_Fe_11.94_O_19_ ceramics, achieved at a thickness of 2.75 mm, exhibited a minimum reflection loss of −14.93 dB within the X-band [15]. Khademi et al. successfully synthesized Ba_1−x_Eu_x_Fe_12_O_19_ powders via the sol–gel approach, revealing that the enhancement in coercivity attributed to Eu doping stems primarily from heightened magnetocrystalline anisotropy. This anisotropy variation was attributed to the transformation of Fe^3+^ ions to Fe^2+^ ions, resulting in a minimum reflection loss of −43 dB in the Ku-band (12–18 GHz) at x = 0.1 [16]. Shi et al. utilized the solid-phase reaction method to synthesize hexagonal lamellar barium ferrite BaNi_x_Zr_x_Fe_12−x_O_19_, achieving a remarkable minimum reflection loss of −60.6 dB and a broad bandwidth of 7.68 GHz in a 2.1 mm-thick sample at x = 0.6. They further demonstrated the tunability of microwave-absorption properties through Ni^2+^-Zr^4+^ co-doping [17]. Feng et al. prepared BaFe_12−x_Co_x_O_19_ via the solid-phase reaction method, achieving a minimum reflection loss of −32.1 dB in a 2 mm-thick sample with x = 0.4. Their findings highlighted the improvement in magnetic properties facilitated by Co ion introduction [18]. As above, numerous studies have focused on single or co-doping at either the barium or iron sites, and the wave-absorbing frequency bands of materials with better comprehensive properties are mostly concentrated in the Ku band. At the same time, limited research has explored the synergistic effects of doping at both sites simultaneously.

In the present work, building upon previous research reported in [19], we chose to deliberately incorporate a specific quantity of Ca ions into the Ba sites of BaM-type ferrites. This approach has been proven to optimally enhance the magnetoelectric properties. Furthermore, considering the influence of Co ions on the magnetocrystalline anisotropy of hexagonal ferrites, as well as their ability to interact with the magnetic moments of adjacent ions through their spin magnetic moments, thereby modulating the overall magnetic properties of the material, we incorporated Co into the Fe sites. The objective of this study was to investigate the subsequent impact on microstructure evolution, phase composition, and magnetic characteristics. Additionally, we aimed to assess the complex permittivity, complex permeability, and ultimately, the microwave-absorption capabilities of the material within the designated frequency band of 1–18 GHz.

## 2. Materials and Methods

BaM-type hexaferrite powders with the general formula BaFe_12_O_19_ (BFO), Ba_0.95_Ca_0.05_Fe_12_O_19_ (BCFO), and Ba_0.95_Ca_0.05_Fe_12−x_Co_x_O_19_ (0 < x ≤ 0.4) (BCFCO) were synthesized by the conventional solid-phase method. The raw materials were selected from BaCO_3_ (99%, Aladdin Scientific Corp., Shanghai, China), Co_3_O_4_ (99%, Aladdin Scientific Corp., Shanghai, China), and Fe_2_O_3_ (99%, Aladdin Scientific Corp., Shanghai, China), and CaCO_3_ (>99%, Aladdin Scientific Corp., Shanghai, China) produced by Aladdin Ltd. For the synthesis of the materials, different stoichiometric ratios of chemicals were used, depending on the target composition. Specifically, BaFe_12_O_19_ was prepared using 1 mole of BaCO_3_ and 6 moles of Fe_2_O_3_. Ba_0.95_Ca_0.05_Fe_12_O_19_ was synthesized by combining 0.95 moles of BaCO_3_, 0.05 moles of CaCO_3_, and 6 moles of Fe_2_O_3_. For Ba_0.95_Ca_0.05_Co_x_Fe_12−x_O_19_ (where x represents the moles of Co), the preparation involved 0.95 moles of BaCO_3_, 0.05 moles of CaCO_3_, x/3 moles of Co_3_O_4_, and (12 − x)/2 moles of Fe_2_O_3_. After mixing the ingredients in stoichiometric ratios, deionized water, zirconia ball-mill beads, and the mixed ingredients were mixed in a ball milling jar in a ratio of 1:1:4 and placed in a planetary ball mill, in which the ball milling rate is 400 rpm for 4 h. The mixture was dried at 110 °C for 12 h and reground. The powder was then placed in a muffle furnace and calcined at 1000 °C for 2 h with a temperature-increase rate of 5 °C/min. The pre-calcined powder was then pelletized and milled for half an hour, with the addition of 5% polyvinyl alcohol (PVA). Toroidal specimens (outside diameter of 7 mm, inside diameter of 3 mm, and thickness of 3~5 mm) were pressed at 20 MPa. Finally, these samples were placed in a muffle furnace and sintered at 1200 °C for 2 h, with a ramp rate of 5 °C/min.

The phase compositions of the samples were analyzed by the X-ray diffractometer (XRD, Ultina IV, Rigaku Corp., Tokyo, Japan) at a voltage of 40 kV and a current of 40 mA, with a testing angle range of 20°–80°, a scanning step of 0.02°, and a scanning speed of 20°/min. The surface morphology was analyzed using a field-emission scanning electron microscope (FE-SEM, S-4700, Hitachi Ltd., Tokyo, Japan), which was operated at a voltage of 15 kV. The magnetic hysteresis loops of the samples were evaluated at room temperature utilizing a vibrating-sample magnetometer (VSM, LakeShore 7404, Lake Shore Cryotronics, Columbus, OH, USA), capable of generating a maximum magnetic field of ±30 kOe.

The permeability and permittivity values were obtained from the *S*_11_ and *S*_21_ parameters measured via the coaxial-line method in 1–18 GHz by a vector network analyzer (VNA, E5071C, Agilent Tech., Santa Clara, CA, USA). The real part (*ε*′) and imaginary part (*ε*″) of the complex permittivity can be calculated using the following formulas:

(1) $$\varepsilon^{\prime }=\frac{c\cdot \left(1+{\left|S_{11}\right|}^{2}-{\left|S_{21}\right|}^{2}\right)}{2\cdot d\cdot {\left|S_{21}\right|}^{2}}$$

(2) $$\varepsilon^{\prime \prime }=\frac{c\cdot tan\left(\theta \right)}{d}$$

where *c* is the speed of light, *d* is the sample thickness, *S*_11_ is the reflection coefficient, *S*_21_ is the transmission coefficient, and *θ* is the phase angle.

Similarly, the real part (*μ*′) and imaginary part (*μ*″) of the complex permeability can be determined:

(3) $$\mu^{\prime }=\frac{1}{{\left|S_{21}\right|}^{2}}\left(\frac{1-{\left|S_{11}\right|}^{2}}{2\cdot {\left|S_{21}\right|}^{2}}\right)$$

(4) $$\mu^{\prime \prime }=\frac{1}{{\left|S_{21}\right|}^{2}}\left(\frac{tan\left(\theta \right)}{2\cdot \left|S_{21}\right|}\right)$$

where *θ* is the phase angle. For this measurement, the sintered ring-shaped specimens were machined to an outer diameter of 7 mm, an inner diameter of 3.04 mm, and a thickness of 2 mm.

The microwave-absorption characteristics of the samples are evaluated by means of reflection loss (*RL*) calculated based on transmission line theory. The calculation formula can be expressed as follows [20]:

(5) $$RL=20lg\left|\frac{Z_{in}-Z_{0}}{Z_{in}+Z_{0}}\right|$$

(6) $$Z_{0}=\sqrt{\frac{\mu_{0}}{\varepsilon_{r}}}$$

(7) $$Z_{in}=Z_{0}\sqrt{\frac{\mu_{r}}{\varepsilon_{r}}}\tanh\left(j\frac{2\pi fd}{c}\sqrt{\mu_{r}\varepsilon_{r}}\right)$$

where *Z_in_*, *Z*_0_, *μ*_0_, *ε*_0_, *μ_r_*, *ε_r_*, and *d* refer to input impedance, free space impedance, permeability of vacuum, permittivity of vacuum, complex magnetic conductance, complex dielectric constant, and material thickness, respectively.

## 3. Results and Discussion

### 3.1. Phase Component

**Figure 1:**
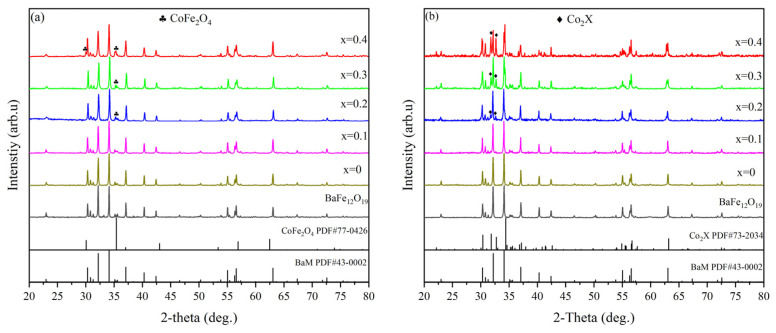
XRD patterns of BaFe_12_O_19_ and Ba_0.95_Ca_0.05_Fe_12−x_Co_x_O_19_ (x = 0, 0.1, 0.2, 0.3, 0.4, respectively) of (**a**) the pre-sintered powders and (**b**) sintered samples.

The XRD patterns of the pre-fired powders calcined at 1000 °C and toroidal specimens sintered at 1200 °C are shown in Figure 1. Through comparison with powder diffraction standard cards, it is observed in Figure 1a that the phase composition of the samples undergoes significant changes with an increasing Co doping amount. For calcined powders with a Co doping amount ranging from x = 0 to 0.2, the primary phase remains the BaM-phase, consistent with the standard card PDF#43-0002. As the doping amount increases to x ≥ 0.3, the spinel phase CoFe_2_O_4_ (PDF#77-0426) gradually increases in the samples. The appearance of CoFe_2_O_4_ is primarily attributed to the increasing chemical reaction between Co_3_O_4_ and Fe_2_O_3_ in the raw material powder, as described by the following reaction equation:

(8) $$2{Co}_{3}O_{4}+6{Fe}_{2}O_{3}\to 6Co{Fe}_{2}O_{4}+O_{2}↑$$

As shown in Figure 1b, the phase composition of the sintered samples also changes with the increase in Co doping amount: in the undoped sample and the samples with doping a amount of x = 0 and 0.1, the main phase is the BaM phase; when the doping amount is x = 0.2, a small amount of Co_2_X phase (PDF#73-2034) was generated; as the doping amount reaches x ≥ 0.3, the amount of the second-phase Co_2_X phase increases significantly, which is attributed to the chemical reaction between the spinel phase CoFe_2_O_4_ and the BaM phase, as described in the following reaction equation:

(9) $$2Ba{Fe}_{12}O_{19}+2Co{Fe}_{2}O_{4}\to {Ba}_{2}{Co}_{2}{Fe}_{28}O_{46}$$

**Figure 2:**
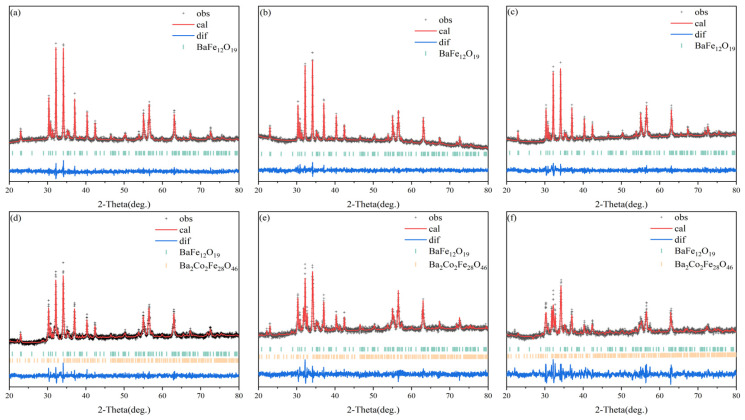
XRD refinement patterns of (**a**) BaFe_12_O_19_ and Ba_0.95_Ca_0.05_Fe_12−x_Co_x_O_19_ with different doping amounts of (**b**) x=0; (**c**) x = 0.1; (**d**) x = 0.2; (**e**) x = 0.3; and (**f**) x = 0.4, respectively.

To further investigate the relative abundance of the BaM and BaX phases within samples containing varying Co concentrations, XRD refinement of the sintered ring samples was conducted, utilizing the GSAS2 software (version 5455). The subsequent outcomes of this refinement process are presented in Figure 2a–f and Table 1, providing a comprehensive illustration of the detailed results. The small Rwp and GOF values in the above XRD refinement data indicate the effectiveness of the refinement process and the reliability of the refinement results. The refinement results show that when the Co doping amount is lower than x = 0.2, all samples are BaM phase, without the existence of the second phase. When the Co addition amount x is equal to or greater than 0.2, the Co_2_X phase appears, and its relative content in the samples increases from 6.7% to 56.3% with the increase in Co addition.

### 3.2. Micromorphology

**Figure 3:**
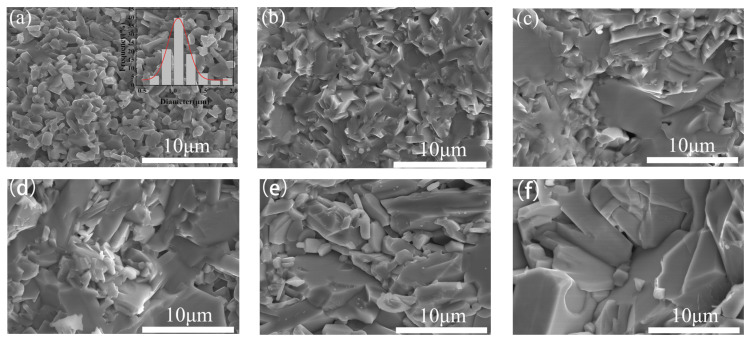
SEM micrographs of (**a**) BaFe_12_O_19_ and Ba_0.95_Ca_0.05_Fe_12−x_Co_x_O_19_ with different doping amounts of (**b**) x = 0; (**c**) x = 0.1; (**d**) x = 0.2; (**e**) x = 0.3; and (**f**) x = 0.4, respectively.

The fracture cross-sectional SEM images of BaFe_12_O_19_ and Ba_0.95_Ca_0.05_Fe_12−x_Co_x_O_19_ ceramics (x = 0, 0.1, 0.2, 0.3, and 0.4, respectively) are presented in Figure 3. BaFe_12_O_19_ in Figure 3a exhibits a uniform grain size distribution, with grains approximately 1 μm in diameter as shown in the insert figure.

Figure 3b vividly displays the effect of calcium ion doping, resulting in enlarged grains with an average size of 2–3 μm and well-defined grain boundaries. This observation is consistent with the literature, which suggests that the incorporation of calcium ions will reduce the sintering temperature of the sample to a certain extent, thereby facilitating grain growth. As shown in Figure 3c–e, the grain size of Ba_0.95_Ca_0.05_Fe_12−x_Co_x_O_19_ ceramics increases with the increasing amount of Co addition. Grain growth may be attributed to the following two factors. Firstly, the introduction of cobalt ions reduces the sintering temperature. Secondly, the increase in Co content promotes the solid-state reaction between the BaM phase and the spinel-phase CoFe_2_O_4_, leading to the formation of a secondary Co_2_X-phase. This phase transition is accompanied by changes in lattice parameters, causing lattice distortion, which, in turn, promotes grain growth to a certain extent. Furthermore, the presence of lattice distortion can also affect the loss mechanism of the sample, thereby influencing the microwave-absorbing properties of the material.

### 3.3. Static Magnetic Properties

**Figure 4:**
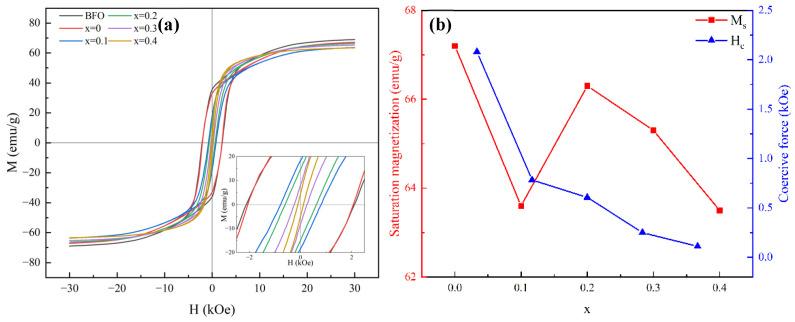
(**a**) The hysteresis loops of BaFe_12_O_19_ and Ba_0.95_Ca_0.05_Fe_12−x_Co_x_O_19_ (x = 0, 0.1, 0.2, 0.3, and 0.4, respectively); (**b**) saturated magnetization strength (Ms) and coercivity (Hc) versus doping amount x.

As shown in Figure 4a, the investigation of hysteresis loops reveals the magnetic properties of BaFe_12_O_19_ and Ba_0.95_Ca_0.05_Fe_12−x_Co_x_O_19_ ceramics (x = 0, 0.1, 0.2, 0.3, and 0.4, respectively) at room temperature. The shape of the hysteresis loop undergoes significant changes with the increase in cobalt ion doping, transforming from the broad and square shape, characteristic of the hard magnetic phase, to the elongated shape, characteristic of the soft magnetic phase. This phenomenon is closely related to the emergence of the second phase, the BaX-phase, as it belongs to the soft magnetic phase, whereas the BaM-phase belongs to the hard magnetic phase. The exchange coupling between these phases can significantly influence the magnetic properties [21]. In hard–soft magnetic composites, if the hysteresis line exhibits a kink phenomenon, it indicates that there is no exchange coupling between the hard magnetic phase and the soft magnetic phase [22]. Samples exhibiting a BaX phase show very smooth hysteresis curves, indicating the presence of effective exchange coupling between the hard magnetic phase (BaM phase) and the soft magnetic phase (Co_2_X phase).

Figure 4b illustrates the variation in saturation magnetization Ms and coercivity Hc of Ba_0.95_Ca_0.05_Fe_12−x_Co_x_O_19_ ceramics with the amount of Co doping. The saturation magnetization of Ba_0.95_Ca_0.05_Fe_12_O_19_ is 69 emu/g, which is very close to the theoretical saturation magnetization of BaM (72 emu/g). As Co doping increases, Ms initially decreases from 69 emu/g to 63.6 emu/g, then rises to 66.3 emu/g, and finally decreases to 63.5 emu/g. A pivotal transition occurs at x = 0.2. For Ba_0.95_Ca_0.05_Fe_12−x_Co_x_O_19_ ceramics with x < 0.2 which have the single phase of BaM, Co ions preferentially occupy the 2a and 12k sites typically held by Fe ions in BaFe_12_O_19_, resulting in reduced overall magnetic moments due to the smaller magnetic moments of Co ions compared to Fe ions, thereby decreasing the saturation magnetization [23,24]. For the sample with x = 0.2, the generation of the secondary phase BaX leads to a diminished population of Co ions in the spin-up positions in the BaM phase, consequently resulting in an increase in the saturation magnetization, to 66.3 emu/g. According to the literature on soft magnetic/permanent magnetic composites, the saturation magnetization of these materials is influenced by various factors, including the relative content of each phase, their individual saturation magnetization values, interactions during the magnetization process, and potential interface effects. For micron-scale soft magnetic/permanent magnetic composites, the primary influencing factors are the relative content and saturation magnetization of each phase. Upon further addition of Co to Ba_0.95_Ca_0.05_Fe_12−x_Co_x_O_19_, a notable increase in the content of the Co_2_X phase can be observed, combining the refined XRD data. Considering that the theoretical saturation magnetization of Co_2_X (57 emu/g) is lower than that of the BaM phase, it becomes evident that, as the Co content increases, the saturation magnetization of the sample decreases gradually [25].

A monotonically decreasing trend in the variation of coercivity is shown in Figure 4b, decreasing from 2107.02 Oe to 111.21 Oe with the increase in doping. Previous research shows that coercivity is influenced by both external and internal factors and is more dependent on external factors, such as grain size, shape, degree of orientation, and distribution [26]. In the present work, the primary attribution for the reduction in coercivity is the emergence of the second phase, Co_2_X, which is inherently a soft magnetic phase with low coercivity [27]. The presence of Co_2_X may mitigate the pinning effect between magnetic domains, enabling them to rotate and rearrange more easily under the influence of an external magnetic field, subsequently reducing the coercivity of the material. Furthermore, coercivity is closely related to grain size, with coercivity inversely proportional to grain size once it exceeds a critical threshold. As the amount of Co doping increases, the grain size expands accordingly, leading to a decrease in coercivity. This reduction in coercivity is recognized as an effective strategy for enhancing microwave-absorption performance [28,29].

### 3.4. Microwave Magneto-Dielectric Properties

**Figure 5:**
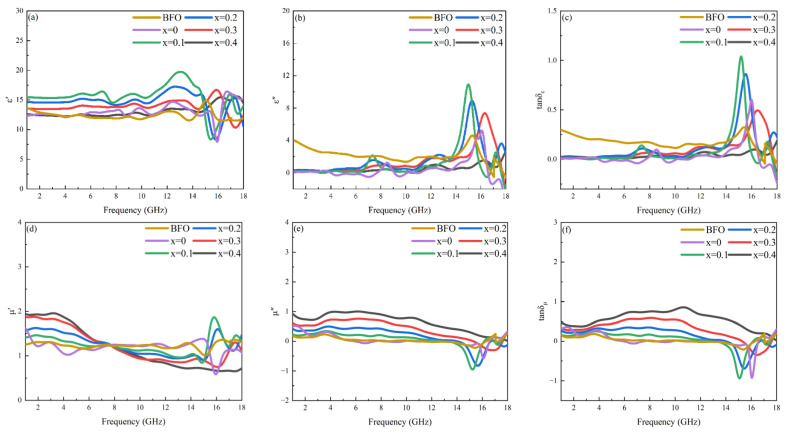
(**a**) Real and (**b**) imaginary parts of complex permittivity, (**c**) dielectric loss tangent, (**d**) real and (**e**) imaginary parts of complex permeability, and (**f**) magnetic loss tangent for BaFe_12_O_19_ and Ba_0.95_Ca_0.05_Fe_12−x_Co_x_O_19_ (x = 0, 0.1, 0.2, 0.3, 0.4) ceramics in the frequency range of 1–18 GHz.

The complex permittivity (ε = ε′–iε″) and complex permeability (μ = μ′–iμ″) in the microwave frequency band are crucial parameters for assessing the performance of absorbing materials. Figure 5 demonstrates the variation in complex permittivity, dielectric loss tangent, complex permeability and magnetic loss tangent of BaFe_12_O_19_ and Ba_0.95_Ca_0.05_Fe_12−x_Co_x_O_19_ (x = 0–0.4) ceramics, with frequency. The real parts of the relative permittivity (ε′) and permeability (μ′) represent the ability of the material to store electric and magnetic energy, respectively. In contrast, the imaginary parts (ε″ and μ″) indicate the tendency of the material to dissipate electric and magnetic energy. Generally, we use the dielectric loss tangent (tanδ_ε_ = ε′/ε″) and the magnetic loss tangent (tanδ_μ_ = μ′/μ″) to describe the energy loss of a material under the action of an electric field and a magnetic field.

Figure 5a–c show that with the doping of cobalt ions, the real part of the dielectric constant of the samples first increases, due to the change in the microstructure of the BaM phase caused by the substitution of Fe ions by Co ions, and then decreases with the emergence of Co_2_X, with a lower relative dielectric constant. At the same time, the imaginary part and dielectric loss tangent decreases, and a broad relaxation peak appears above 14 GHz. The relaxation polarization phenomenon may be related to the compositional inhomogeneity caused by doping, and may also be closely correlated to the difference in grain size [30,31]. Shaikh’s model indicates that the permittivity of micron-scale dielectric materials is inversely proportional to the grain size. As the doping concentration increases, the grain size of Ba_0.95_Ca_0.05_Fe_12−x_Co_x_O_19_ ceramics gradually increases, and the relationship between permittivity and grain size is consistent with this model [32].

The variation in the real, imaginary components and magnetic loss tangent of the sample’s permeability with frequency is depicted in Figure 5d–f, respectively. The real part of the permeability diminishes as the frequency increases, within the range of 1–14 GHz. Furthermore, as the amount of Co doping increases, the rate of this decrease accelerates. In the high-frequency range of 14–18 GHz, the permeability of the samples undergoes notable changes. The imaginary component of the samples’ complex permeability and magnetic loss tangent displays a relaxation-type curve, featuring resonance absorption peaks resulting from intrinsic magnetic anisotropy. Typically, ferrites exhibit two distinct resonance mechanisms in alternating electromagnetic fields: natural resonance and domain-wall resonance. In polycrystalline ferrites, a single magnetic domain’s size is at the sub-micron scale, whereas the particle size in this study significantly exceeds that of a single domain, allowing for the coexistence of these two resonance mechanisms. Predominantly, the resonance absorption peak observed in the microwave frequency band originates from natural resonance. This natural resonance peak enables the imaginary part of the permeability to sustain a high value across a broad frequency spectrum, showcasing exceptional magnetic loss performance within this range. Upon increasing the doping of Co ions, notably when the doping level x reaches or surpasses 0.2, the imaginary part of the permeability undergoes a marked enhancement. This suggests that the emergence of the secondary phase Co_2_X positively contributes to the magnetic loss properties of the samples. It can be noted that μ″ assumes negative values at x = 0, 0.1, and 0.2, which is a phenomenon commonly observed in materials with high loss and conductivity, such as Fe_3_O_4_/SnO_2_ nanorods, multi-walled carbon nanotubes, and various composites [33].

To clarify the specific type of magnetic loss studied in the present work, we calculated the eddy-current loss (*C*_0_) value of the samples. The magnetic loss of ferrites mainly arises from exchange resonance, natural resonance, domain-wall resonance, hysteresis loss, and eddy-current loss. Typically, in the frequency range of 1–18 GHz, domain-wall resonance and hysteresis loss are considered negligible. This is attributed to the fact that hysteresis loss is the result of irreversible magnetization in high magnetic fields, while domain-wall resonance manifests itself in the megahertz frequency range [34]. Exchange resonance, on the other hand, generally requires material particle sizes within 100 nm, which is far exceeded by the hexaferrite sizes prepared in this study, thus contributing less to magnetic loss. The eddy-current loss factor can be expressed by Equation (10):

(10) $$C_{0}=\mu^{\prime \prime }{\left(\mu^{\prime }\right)}^{-2}f^{-1}$$

**Figure 6:**
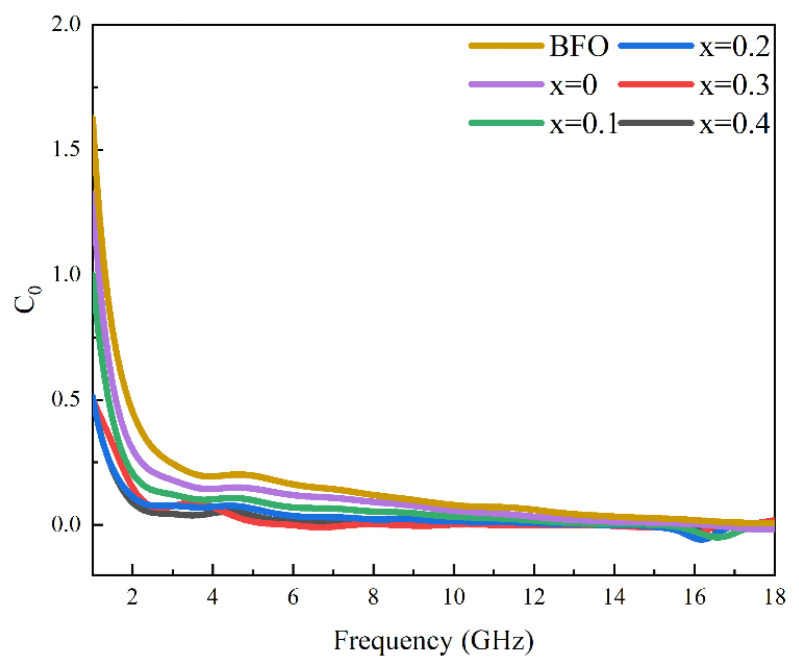
Eddy-current loss for BaFe_12_O_19_ and Ba_0.95_Ca_0.05_Fe_12−x_Co_x_O_19_ (x = 0, 0.1, 0.2, 0.3, 0.4).

When *C*_0_ changes very little with frequency, gradually approaching a straight line, it indicates that the primary magnetic-loss mechanism of the ferrite in this frequency range originates from eddy-current loss. As shown in Figure 6, the variation of the curve at lower frequencies (1–5 GHz) can be attributed to natural resonance, and as the frequency increases, the curve tends to stabilize, suggesting that eddy-current loss has little impact on magnetic loss. In summary, the predominant mechanism governing the absorption of electromagnetic waves by BaFe_12_O_19_ and Ba_0.95_Ca_0.05_Fe_12−x_Co_x_O_19_ (x = 0–0.4) ceramics originates from magnetic loss, with natural resonance playing a pivotal role in contributing to this loss.

### 3.5. Microwave-Absorption Properties

**Figure 7:**
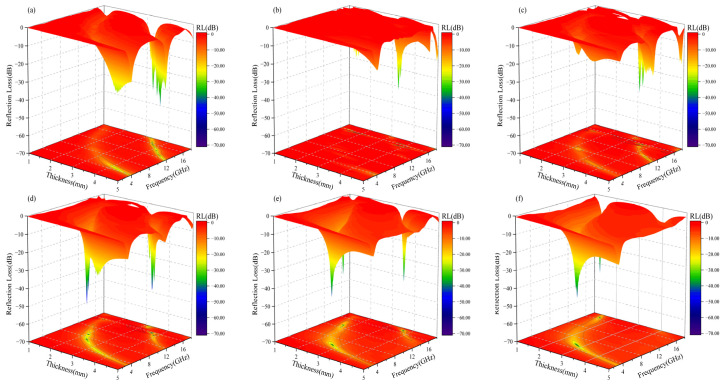
Contour plots of 3D reflection loss for BaFe_12_O_19_ and Ba_0.95_Ca_0.05_Fe_12−x_Co_x_O_19_ ceramics in the frequency range of 1–18 GHz and 1–5 mm thickness (**a**) BFO; (**b**) x = 0; (**c**) x = 0.1; (**d**) x = 0.2; (**e**) x = 0.3; (**f**) x = 0.4.

**Figure 8:**
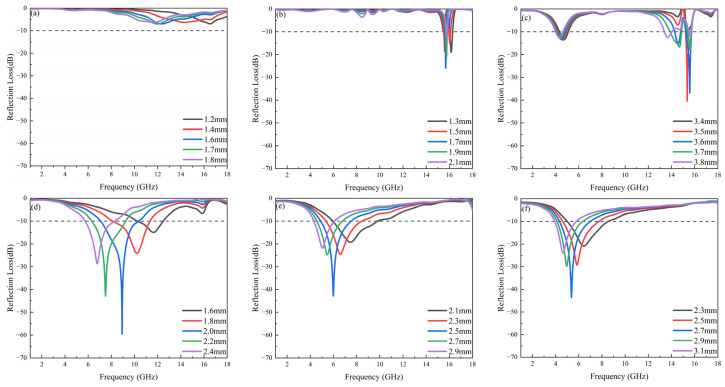
Reflection-loss curves of samples: (**a**) BaFe_12_O_19_ and Ba_0.95_Ca_0.05_Fe_12−x_Co_x_O_19_ with doping amounts of (**b**) x = 0; (**c**) x = 0.1; (**d**) x = 0.2; (**e**) x = 0.3; and (**f**) x = 0.4, in the frequency range of 1–18 GHz. The dotted line indicates a reflection loss of −10 dB.

Figure 7 and Figure 8 demonstrate the microwave-absorption properties of BFO and BCFCO ceramics (x = 0.1, 0.2, 0.3, 0.4) at different thicknesses. It can be observed from Figure 7 that BFO exhibits favorable wave-absorbing properties only when the thickness exceeds 3.5 mm. After Ca ion doping, BCFO achieves a certain effective absorption in the Ku-band across a thickness range of 1–5 mm, but it attains relatively high minimum reflection loss only when the thickness exceeds 3.5 mm, with a narrow effective absorption bandwidth (<1 GHz). With Co ion co-doping, the microwave-absorption characteristics of BCFCO (x = 0.1–0.4) samples improve significantly, and the minimum reflection-loss peak shifts towards lower frequencies as the doping content increases: from the Ku-band at x = 0.1 to the C-band at x = 0.4. The x = 0.1 sample has a thicker optimal matching thickness, but achieves a substantial minimum reflection loss in the Ku-band, albeit with a still narrow effective absorption bandwidth. Samples with x ≥ 0.2 exhibit excellent microwave-absorption performance. Some studies suggest that ferrites with planar anisotropy can achieve wider effective absorption bandwidths at lower frequencies with thinner matching thicknesses, while samples with c-axis anisotropy exhibit good absorption performance at high frequencies only, with larger matching thicknesses [35]. Notably, BaFe_12_O_19_ is renowned for its large uniaxial magnetocrystalline anisotropy field. In contrast to other X-phase ferrites exhibiting uniaxial anisotropy at room temperature, the magnetization cone of Co_2_X forms a 74° angle with the c-axis [36]. Therefore, the presence of conical anisotropic Co_2_X in BCFCO modulates the microwave-absorption properties of BaM, enabling wider effective absorption bandwidths at lower frequencies, with thinner matching thicknesses. Notably, the x = 0.2 sample achieves a minimum reflection loss of −59.5 dB and an effective absorption bandwidth of 3.31 GHz (7.07–10.38 GHz) at a very thin thickness of 2 mm. As the Co ion doping content further increases, the matching thickness of the samples slightly enlarges. This phenomenon can be explained by solving the 1/4-wavelength-matching equation, as follows:

(11) $$d=\frac{n\lambda }{4}=\frac{nc}{4f_{m}\sqrt{\left|\mu_{r}\right|\left|\varepsilon_{r}\right|}}$$

where *d* represents the matching thickness, f_m_ is the frequency, *λ* is the electromagnetic wave wavelength, and c is the speed of light. When the matching thickness of the absorbing material satisfies odd multiples of 1/4 wavelength, one or more peaks in the reflection-loss curve occur. According to Equation (11), as the dielectric constant and permeability of the material increase, the matching thickness decreases, correspondingly. BCFCO samples with x = 0.1, 0.2, and 0.3 possess larger dielectric constant and permeability, thus exhibiting smaller matching thicknesses. Conversely, BFO and BCFO have lower dielectric constants and permeabilities, resulting in larger matching thicknesses. Although the x = 0.4 sample has a lower dielectric constant, its highest permeability at lower frequencies leads to a relatively smaller matching thickness.

**Figure 9:**
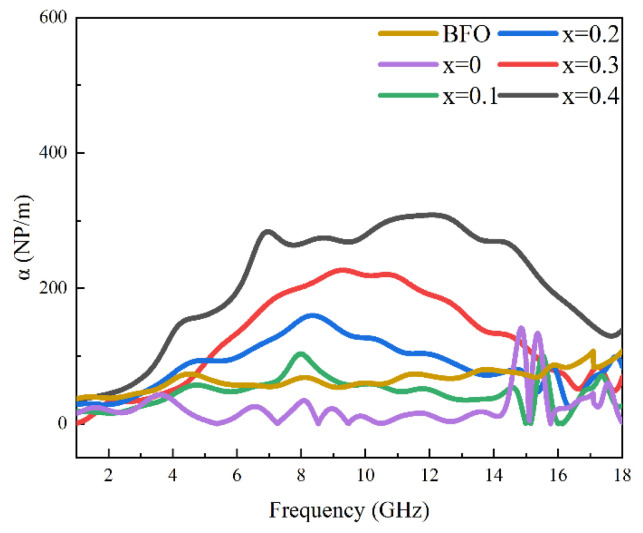
Microwave attenuation coefficients of BaFe_12_O_19_, Ba_0.95_Ca_0.05_Fe_12_O_19_ and Ba_0.95_Ca_0.05_Fe_12−x_Co_x_O_19_ (x = 0.1, 0.2, 0.3, 0.4) in the frequency range of 1–18 GHz.

In addition to good impedance matching characteristics to ensure that incident electromagnetic waves can smoothly enter the absorbing material without much reflection on the surface, good absorbing materials must also have strong attenuation ability for electromagnetic waves, so that they can quickly convert as much as possible of the electromagnetic wave entering the absorbing material into thermal energy, and dissipate it. This attenuation ability can be evaluated by the attenuation constant *α* [37]:

(12) $$\alpha =\left(\frac{\sqrt{2}\pi f}{c}\right)\sqrt{\left(\mu^{\prime \prime }\varepsilon^{\prime \prime }-\mu^{\prime }\varepsilon^{\prime }\right)+\sqrt{\left(\mu^{\prime \prime }\varepsilon^{\prime \prime }-\mu^{\prime }\varepsilon^{\prime }\right)+{\left(\mu^{\prime \prime }\varepsilon^{\prime }+\mu^{\prime }\varepsilon^{\prime \prime }\right)}^{2}}}$$

where *c* refers to the speed of light. Figure 9 shows the variation in the attenuation coefficient with frequency for the BFO, BCFO, and BCFCO (x = 0.1–0.4) samples. It can be seen that after Co doping, the attenuation coefficient of BCFCO samples, especially those with x ≥ 0.2, increases significantly, indicating that the presence of Co_2_X plays a significant role in enhancing the decay ability of the samples. Table 2 provides a comparison of the research findings of the microwave-absorbing properties of M-type hexagonal ferrites. The results of the present work exhibit certain advantages in microwave-absorbing properties and tunable absorption bands, thus providing more options and flexibility for microwave-absorption applications in different spectral ranges. This is of great significance for the development and improvement of microwave-absorbing materials that meet different application requirements.

## 4. Conclusions

The BaFe_12_O_19_ and Ba_0.95_Ca_0.05_Fe_12−x_Co_x_O_19_ (x = 0~0.4) hexaferrites were successfully synthesized by the solid-state reaction method. The effects of Co substitution on the phase composition, microstructure, magnetic properties, and microwave-absorption capabilities of this ferrite system were investigated. The introduction of a small amount of Co ions (x < 0.2) reduced the sintering temperature of the samples and promoted grain growth. When the doping amount of Co ions reached x ≥ 0.2, X-ray diffraction (XRD) analysis showed that, in addition to the BaM phase, the Co_2_X phase also appeared in the samples. As the amount of Co ions and the second-phase X increased, the saturation magnetization of the samples decreased slightly, from 69 emu/g to 63.5 emu/g, while the coercivity decreased from 2107.02 Oe to 111.21 Oe. This significant decrease was related to grain size and the soft magnetic properties of Co_2_X. Simultaneously, the introduction of Co_2_X exhibited significant advantages in terms of the microwave-absorption performance and tunable-absorption frequency bands of the samples. For a sample with a thickness of 2.0 mm and a doping level of x = 0.2, a minimum reflection loss of −59.5 dB was achieved at 8.92 GHz, with an effective absorption bandwidth of 3.31 GHz (7.07–10.38 GHz). This study indicates that this system of composite materials has the potential to become a candidate material for future microwave-absorption applications.

## Figures and Tables

**Table 1:** XRD refinement data of BaFe_12_O_19_ with Ba_0.95_Ca_0.05_Fe_12−x_Co_x_O_19_ (x = 0, 0.1, 0.2, 0.3, 0.4, respectively).

Samples	BaM-Phase (%)	Co_2_X-Phase (%)	Rwp (%)	GOF
BaFe_12_O_19_	100	0	2.48	1.16
x = 0	100	0	2.48	1.08
x = 0.1	100	0	2.41	1.10
x = 0.2	93.3	6.7	2.34	1.14
x = 0.3	67.2	22.8	2.44	1.19
x = 0.4	43.7	56.3	3.44	1.64

**Table 2:** Comparison of absorption performance of M-type hexagonal ferrites.

Samples	Thickness (mm)	RL_min_ (dB)	Bandwidth (GHz)	Absorption Range (GHz)	Frequency (GHz)	Reference
BaZr_1.2_Fe_10.8_O_19_	5	−30.2	2.46	-	16.75	[38]
BaFe_10.6_Co_0.7_Ti_0.7_O_19_	3.3	−25.15	0.94	-	9.54	[39]
BaFe_11.94_La_0.06_O_19_	2.75	−14.93	-	-	-	[15]
x = 0.2	2.0	−59.5	3.31	7.07–10.38	8.9	This work
x = 0.3	2.5	−42.9	2.63	4.9–7.53	5.98	This work

## Data Availability

The authors confirm that the data supporting the findings of this study are available within the article.

## References

[1] Cao M., Qin R., Qiu C., Zhu J. (2003). Matching design and mismatching analysis towards radar absorbing coatings based on conducting plate. Mater. Des..

[2] Li Y., Yang H.-J., Yang W.-G., Hou Z.-L., Jin J.-B., Yuan J., Cao M.-S. (2013). Structure, ferromagnetism and microwave absorption properties of La substituted BiFeO_3_ nanoparticles. Mater. Lett..

[3] Jasrotia R., Prakash J., Himanshi, Thakur N., Raj K., Kandwal A., Sharma P. (2023). Advancements in doping strategies for enhancing applications of M-type hexaferrites: A comprehensive review. Prog. Solid State Chem..

[4] Wang D., Wang M., Liu F., Cui Y., Zhao Q., Sun H., Jin H., Cao M. (2015). Sol–gel synthesis of Nd-doped BiFeO_3_ multiferroic and its characterization. Ceram. Int..

[5] Mohammad F., Huma F., Ali K. (2022). Hydrothermal synthesisi and microwave absorption properties of reduced graphene oxide/BaCo_0,2_ Ti_0.2_Fe_1.6_O_19_ nanocomposites. J. Magn. Magn. Mater..

[6] Wang Y., Luo Z., Hong R. (2011). Microstructure and microwave absorption properties of Fe_3_O_4_/dextran/SnO_2_ multilayer microspheres. Mater. Lett..

[7] Huang X., Zhang J., Wang L., Zhang Q. (2012). Simple and reproducible preparation of barium hexagonal ferrite by adsorbent combustion method. J. Alloys Compd..

[8] Liu J., Ma T., Tong H., Luo W., Yan M. (2010). Electromagnetic wave absorption properties of flaky Fe-Ti-Si-Al nanocrystalline composites. J. Magn. Magn. Mater..

[9] Singh C., Narang S.B., Hudiara I., Sudheendran K., Raju K.J. (2008). Complex permittivity and complex permeability of Sr ions substituted Ba ferrite at X-band. J. Magn. Magn. Mater..

[10] Shao N., Li J., Che S., Zheng J., Qiao L., Ying Y., Yu J., Li W. (2023). L and S band microwave absorption properties of Z-type hexaferrite Ba_3_Co_2_Fe_24_O_41_ synthesized at low temperature. J. Alloys Compd..

[11] Yang H., Dai J., Liu X., Lin Y., Wang F., Liu P. (2017). Synthesis and enhanced microwave absorption properties of PVB/Co_2_Z/RGO layered composite. J. Alloys Compd..

[12] Zhang B., Feng Y., Xiong J., Yang Y., Lu H. (2006). Microwave-absorbing properties of de-aggregated flake-shaped carbonyl-iron particle composites at 2–18 GHz. IEEE Trans..

[13] Wei C.-Y., Shen X.-Q., Song F.-Z. (2012). Double-layer microwave absorber of nanocrystalline strontium ferrite and iron microfibers. Chin. Phys. B.

[14] Praveena K., Sadhana K., Liu H.-L., Bououdina M. (2017). Microwave absorption studies of magnetic sublattices in microwave sintered Cr^3+^ doped SrFe_12_O_19_. J. Magn. Magn. Mater..

[15] Goel S., Bala M., Garg A., Shivling V., Tyagi S. (2020). Lanthanum doped barium hexaferrite nanoparticles for enhanced microwave absorption. Mater. Today Proc..

[16] Khademi F., Poorbafrani A., Kameli P., Salamati H. (2012). Structural, magnetic and microwave properties of Eu-doped barium hexaferrite powders. J. Supercond. Nov. Magn..

[17] Shi K., Li J., He S., Bai H., Hong Y., Wu Y., Jia D., Zhou Z. (2019). A superior microwave absorption material: Ni^2+^-Zr^4+^ Co-Doped barium ferrite ceramics with large reflection loss and broad bandwidth. Cur. Appl. Phys..

[18] Feng G., Zhou W., Deng H., Chen D., Qing Y., Wang C., Luo F., Zhou Y. (2019). Co substituted BaFe_12_O_19_ ceramics with enhanced magnetic resonance behavior and microwave absorption properties in 2.6–18 GHz. Ceram. Int..

[19] Kumar S., Supriya S., Pandey R., Pradhan L., Singh R., Kar M. (2018). Effect of lattice strain on structural and magnetic properties of Ca substituted barium hexaferrite. J. Magn. Magn. Mater..

[20] Wang M., Lin Y., Yang H., Qiu Y., Wang S. (2020). A novel plate-like BaFe_12_O_19_@MoS_2_ core-shell structure composite with excellent microwave absorbing properties. J. Alloys Compd..

[21] Pahwa C., Narang S., Sharma P. (2020). Composition dependent magnetic and microwave properties of exchange-coupled hard/soft nanocomposite ferrite. J. Alloys Compd..

[22] Shen X., Song F., Yang X., Wang Z., Jing M., Wang Y. (2015). Hexaferrite/a-iron composite nanowires: Microstructure, exchange-coupling interaction and microwave absorption. J. Alloys Compd..

[23] Chen J., Liu Y., Yin Q. (2022). Computational and experimental study on cation distribution of cobalt substituted barium hexaferrites BaFe_12-x_Co_x_O_19_ (x = 0, 0.3, 0.6, 0.9) for circulator applications. J. Alloys Compd..

[24] Yang Y., Wang F., Shao J., Huang D., Trukhanov A., Trukhanov S. (2018). Preparation of Al^3+^-Co^2+^ co-substituted M-type SrCaNd hexaferrites and their controlled magnetic properties. AIP Adv..

[25] Tran N., Choi Y., Phan T., Yang D., Lee B. (2019). Electronic structure and magnetic and electromagnetic wave absorption properties of BaFe_12-x_Co_x_O_19_ M-type hexaferrites. Curr. Appl. Phys..

[26] Liu C., Zhang Y., Tang Y., Wang Z., Ma N., Du P. (2017). The tunable magnetic and microwave absorption properties of the Nb^5+^–Ni^2+^ co-doped M-type barium ferrite. J. Mater. Chem. C.

[27] Kamishima K., Hosaka N., Kakizaki K., Hiratsuka N. (2011). Crystallographic and magnetic properties of Cu_2_X, Co_2_X, and Ni_2_X hexaferrites. J. Appl. Phys..

[28] Panagiotopoulos I. (2011). A simple approach to the First Order Reversal Curves (FORC) of two-phase magnetic systems. J. Magn. Magn. Mater..

[29] Algarou N., Slimani Y., Almessiere M., Alahmari F., Vakhitov M., Klygach D., Trukhanov S., Trukhanov A., Baykal A. (2020). Magnetic and microwave properties of SrFe_12_O_19_/MCe_0.04_Fe_1.96_O_4_ (M = Cu, Ni, Mn, Co and Zn) hard/soft nanocomposites. J. Mater. Res. Technol..

[30] Liu C., Hao Y., Zheng S., Fang G., Li J., Tao S., Zhang Y., Du P. (2023). Abating dopant competition between dual high-valence ions in single-phased barium ferrite towards ultra-broad microwave absorption. J. Mater. Chem. C.

[31] Batoo K., Kumar S., Lee C., Alimuddin (2009). Finite size effect and influence of temperature on electrical properties of nanocrystalline Ni–Cd ferrites. Curr. Appl. Phys..

[32] Shaikh A., Vest R., Vest G. Dielectric properties of ultra fine grained BaTiO_3_. Proceedings of the IEEE International Symposium on Applications of Ferroelectrics.

[33] Hou Z., Zhang M., Kong L., Fang H., Li Z., Zhou H., Jin H., Cao M. (2013). Microwave permittivity and permeability experiments in high-loss dielectrics: Caution with implicit Fabry-Pérot resonance for negative imaginary permeability. Appl. Phys. Lett..

[34] Liu P., Gao S., Wang Y., Huang Y., Wang Y., Luo J. (2019). Core–shell CoNi@graphitic varbon decorated on B,N-codoped hollow carbon polyhedrons toward lightweight and high-efficiency microwave attenuation. ACS Appl. Mater. Interfaces.

[35] Jin L. (2022). Research on Microwave Absorbing Properties of Doped Co_2_Z Type Hexagonal Ferrite and Its Composites. Master’s Thesis.

[36] Robert C. (2012). Hexagonal ferrites: A review of the synthesis, properties and applications of hexaferrite ceramics. Prog. Mater. Sci..

[37] Goel S., Garg A., Baskey H., Pandey M., Tyagi S. (2021). Studies on dielectric and magnetic properties of barium hexaferrite and bio-waste derived activated carbon composites for X-band microwave absorption. J. Alloys Compd..

[38] Deng L., Zhao Y., Xie Z., Liu Z., Tao C., Deng R. (2018). Magnetic and microwave absorbing properties of low-temperature sintered BaZr_x_Fe_12−x_O_19_. RSC Adv..

[39] Narang S.B., Kaur P., Bahel S., Singh C. (2016). Microwave characterization of Co-Ti substituted barium hexagonal ferrites in X-band. J. Magn. Magn. Mater..

